# Supplementary material for: Intracranial Assessment of Androgen Receptor Antagonists in Mice Bearing Human Glioblastoma Implants
Source: Int J Mol Sci. 2023 Dec 26;25(1):332. doi: 10.3390/ijms25010332 (PMC10779261; doi:10.3390/ijms25010332)
Supplement: Supplementary file 1 [file ijms-25-00332-s001.zip › ijms-2768619-supplementary.pdf]

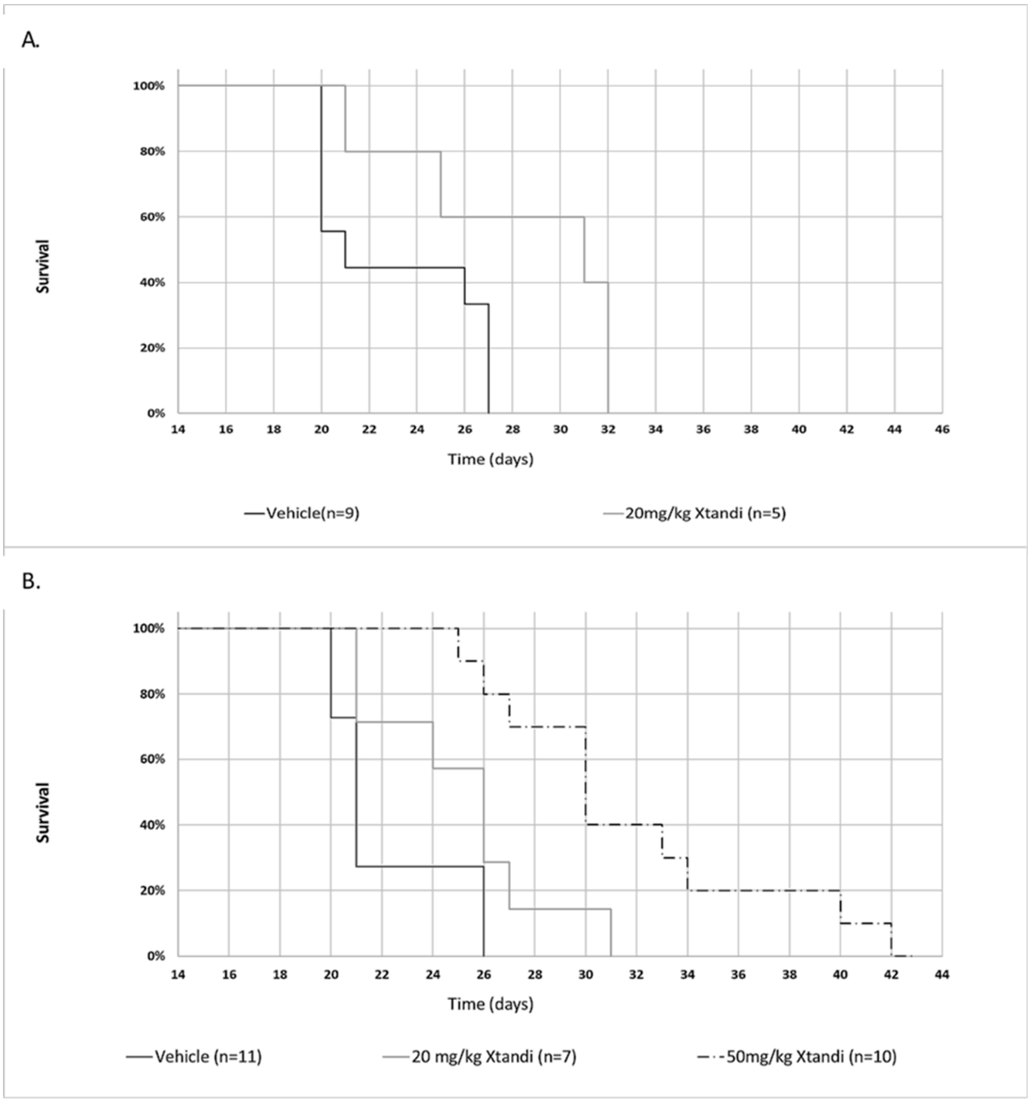

Supplementary Figure S1. Kaplan-Meier Analysis of Nude Mice with Intracranial U87MG Human Glioblastoma Tumors Treated with enzalutamide (XTANDI).

Nude mice with intracranial U87MG glioblastoma tumors were treated 5 times weekly with XTANDI or vehicle control. The survival of three groups was assessed: vehicle-treated mice (solid black line), mice treated with 20 mg/kg XTANDI (solid grey line), and mice treated with 50 mg/kg XTANDI (dash-dotted line).

A. an experiment involving a vehicle group (n = 9) and a group treated with 20 mg/kg XTANDI (n = 5), mice treated with XTANDI® (enzalutamide) at a dosage of 20 mg/kg exhibited a significant extension in lifespan compared to the vehicle-treated group (log-rank test; $\chi^2 = 1.97$, p = 0.0484).

B. an experiment comprising three study groups: vehicle (n = 11), 20 mg/kg XTANDI (n = 7), and 50 mg/kg XTANDI (n = 10), mice treated with XTANDI® at dosages of 20 mg/kg and 50 mg/kg demonstrated a significant extension in lifespan compared to the control group receiving vehicle treatment (log-rank test; z = 1.94, $p = 0.05$ and z = 3.91, $p < 0.001$, respectively).
